# User-Involvement in the Development of a Culturally Sensitive Intervention in the Safe Pregnancy Study to Prevent Intimate Partner Violence

**DOI:** 10.1177/1077801220954274

**Published:** 2020-09-28

**Authors:** Eva Marie Engebakken Flaathen, Mirjam Lukasse, Lisa Garnweidner-Holme, Jeanette Angelshaug, Lena Henriksen

**Affiliations:** 1 Oslo Metropolitan University, Norway; 2 University of South-Eastern Norway, Norway; 3 Oslo University Hospital, Norway

**Keywords:** intimate partner violence, pregnancy, antenatal care, interventions, culture-sensitivity, user involvement, mHealth technology

## Abstract

Intimate partner violence (IPV) during pregnancy has negative health impacts on the woman and the fetus. There is a lack of evidence supporting effective interventions to prevent IPV during pregnancy. This user-involvement study was conducted to get feedback on a culturally sensitive, tablet intervention containing questions about violence and safety-behaviors and a video promoting safety behaviors. This resulted in important feedback on the intervention content. Our findings show that women are in favor of disclosing IPV via a tablet. They suggested ways to address barriers for disclosure, such as safeguarding anonymity and creating a trustful relationship with the midwife.

## Introduction

### Intimate Partner Violence (IPV)

Intimate partner violence is considered a serious social and public health problem, with one in three women having experienced physical and/or sexual violence by an intimate partner some time during their life (García-Moreno et al., 2013). Pregnancy does not protect women against violence. On the contrary, pregnancy is recognized as a vulnerable period for IPV because it is a time of change in physical, emotional, social, and economic demands and needs (Van Parys et al., 2014).

A recent meta-analysis of IPV during pregnancy, which included 92 studies from 23 countries, reported that the prevalence of physical, sexual, and emotional abuse was 13.8%, 8.0%, and 28.4%, respectively (James et al., 2013). In Norway, the prevalence of IPV during pregnancy ranges from 1-5% (Henriksen et al., 2014; Hjemdal & Engnes, 2009; Lukasse et al., 2014; Sorbo et al., 2013). These numbers are comparable with a longitudinal cohort study from Sweden reporting that 2-5% of 1,573 women experienced violence during pregnancy (Finnbogadottir et al., 2014). Finnbogadottir and Dykes (2016) found that the prevalence increased in the early postnatal period.

Violence during pregnancy is associated with adverse complications, such as premature contractions, miscarriage, premature birth, still birth, and low birth weight (Alhusen et al., 2015; Brownridge et al., 2011; Henriksen et al., 2013; Hill et al., 2016). In addition, it may affect motherhood and the way women connect and interact with their babies (Hooker et al., 2016).

### Interventions to Prevent IPV in Antenatal Care

Antenatal care is considered a “window of opportunity” to address IPV because women are in regular contact with health professionals throughout the pregnancy (Devries et al., 2010). Pregnancy is an important context for safety planning as their child’s well-being and safety is a priority to many abused women (Vatnar & Bjorkly, 2010). In Norway, almost all pregnant women attend antenatal care for checkups, which is a free and well-integrated service in the public health system (Helsedirektoratet, 2019).

Recommended interventions for IPV in primary care settings involve questions about violence, safety-planning strategies, and help-seeking strategies (Burnett & Bacchus, 2016; J. M. McFarlane et al., 2006). The evidence regarding how to assess and intervene to prevent IPV during pregnancy and the new born period is inconclusive (Jahanfar et al., 2013; Kiely et al., 2010; Pallitto et al., 2016; Tiwari et al., 2005) and there is lack of evidence regarding the effectiveness of interventions aiming to prevent IPV during pregnancy (Van Parys et al., 2014).

Screening for IPV in antenatal care might increase identification of violence (O’Doherty et al., 2014). Norwegian guidelines (Helsedirektoratet, 2014) in antenatal care strongly recommend health professionals routinely ask women about experiences of IPV. Health professionals report barriers to assess for IPV in face-to-face clinical settings, such as their discomfort with IPV questioning, fear of offending women, lack of communication skills, and uncertainty about management after disclosure (Eustace et al., 2015; Henriksen et al., 2017; Hjemdal & Engnes, 2009; S. Sprague et al., 2012). In addition, communication about IPV seems to be especially challenging when meeting with a multicultural patient, indicating the need for culturally sensitive communication strategies (Hawkins et al., 2008; Kreuter & McClure, 2004). Abused women also describe difficulties managing disclosure in face-to-face interventions: they worry about confidentiality and judgmental responses (Byrskog et al., 2016; Edin et al., 2010; G. Feder et al., 2009; Garnweidner-Holme et al., 2017; Stenson et al., 2001).

Mobile health technology (mHealth) such as mobile phones, tablets, and other wireless computing devices have the potential to overcome some of the barriers regarding face-to-face interventions (Bacchus et al., 2016; World Health Organization [WHO], 2011). The technology supports Audio Computer Assisted Self Interviews (ACASI) that tend to yield higher rates of IPV disclosure than face-to-face interventions (Klevens et al., 2012). Online interventions can promote confidentiality and privacy, be accessed anonymously, and may help to standardize the way IPV assessments and interventions are delivered (Bacchus et al., 2016; Hegarty et al., 2015). Women who do not want to disclose violence face-to-face with health professionals may welcome self-completion of IPV screening (Taft et al., 2015). How health professionals bring their own values and past IPV trauma into the screening process can be a positive or negative element of face-to-face disclosure depending on how it is managed by them (C. Sprague et al., 2017).

### The Safe Pregnancy Study

The Safe Pregnancy study is a randomized controlled trial (RCT) to test the effectiveness of a tablet-based, culturally adapted intervention in antenatal care aiming to promote safety behaviors and prevent IPV among pregnant Norwegian, Pakistani, and Somali women (Henriksen et al., 2019). The RCT is being carried out in a routine antenatal care setting at 19 mother and child health centers (MCHC) in south-eastern Norway. The recruitment of participants took place between January 2018 and June 2019, but the study is ongoing with follow-up. The intervention consists of questions and a video about violence and safety behaviors (Henriksen et al., 2019). The following questions and tools about violence have been used in the intervention:

A modified version of the Abuse Assessment Screen (AAS), a five-item screening tool for violence in pregnancy, is part of the baseline questionnaire (Henriksen et al., 2019; Moonesinghe et al., 2004; Rabin et al., 2009). Women who screen positive for IPV are asked to complete the Composite Abuse Scale R-SF (CAS), an instrument containing 15 questions about physical, psychological, and sexual violence (Ford-Gilboe et al., 2016). The safety behaviors have been modified from McFarlane’s safety behavior checklist (Henriksen et al., 2019; J. McFarlane et al., 2004).

The intervention video lasts for 7 min and consists of storytelling combined with digital content, including pictures, images, sound, and video, focusing on information about the definition and types of IPV, the cycle of abuse, IPV during pregnancy and the health consequences, help-seeking strategies, and safety-promoting behaviors. According to Gidman (2013), digital storytelling is a strategy to empower people and facilitate learning.

The Safe Pregnancy study is a culturally sensitive intervention aiming to promote safety behaviors during pregnancy. The intervention materials have been professionally translated into Norwegian, English, Urdu, and Somali. With help from the current user-involvement study (UIS), the intervention has been culturally adapted by involving women with Norwegian, Pakistani, and Somali backgrounds in the development process.

### The UIS

The UIS presented in this article was an iterative development process that engaged users in conceptualization, design, and final production of the tablet-based intervention (LeRouge et al., 2013). User involvement helps to gain deeper knowledge about cultural differences and create culturally sensitive interventions (Kreuter et al., 2003; Resnicow et al., 1999; Van Parys et al., 2014). The specific aim of the UIS was to get feedback from participants on questions about violence and safety behaviors and to receive information about the content in the intervention video. The users’ feedback provided important insights into the process of developing a useful and culturally sensitive, tablet-based intervention aiming to prevent IPV in antenatal care.

## Method

### Recruitment and Participants

The recruitment of participants took place over 9 months in south eastern Norway starting in May 2017. Participant inclusion criteria for individual interviews were largely identical to those in the Safe Pregnancy study to facilitate similarities in the two study populations (Henriksen et al., 2019). Norwegian, Pakistani, and Somali women, 18 years and above, with or without former experiences of IPV, were recruited. Selection and recruitment of participants followed a purposive and snowball sampling approach. Women with a history of IPV were recruited through shelters. Women with immigrant backgrounds, with and without a history of IPV, were difficult to recruit; therefore, snowball sampling facilitated recruiting these participants. Focus group participants were recruited from two shelters. The recruitment finished when richness and saturation of data from individual and focus group participants were reached (Braun & Clarke, 2006).

Sixteen women were recruited for individual interviews. The women’s ages ranged from 22-47 years. Table 1 summarizes the women’s background characteristics. The focus groups comprised between two and three participants, including four women and one man. The participants originated from Norway (*n* = 3), India (*n* = 1), and Iran (*n* = 1), were skilled professionals reporting high education levels, and were community workers, social workers, and teachers.

**Table 1.** Individual Interviews: Women Characteristics (*n* = 16).

Participant	Country of birth	Ethnic background	Parity and age of the youngest child (years)	Higher education	Experienced violence
1	Norway	—	0	Yes	No
2	Norway	—	3 (1)	No	Yes
3	Norway	—	1 (1)	No	Yes
4	Pakistan	—	1 (3)	Yes	Yes
5	Norway	—	1 (4)	Yes	Yes
6	Pakistan	—	2 (21)	No	Yes
7	Norway	Pakistani	1 (4)	No	No
8	Norway	Somali	0	Yes	No
9	Norway	—	1 (1)	Yes	No
10	Somalia	—	0	No	No
11	Norway	—	1 (7)	Yes	No
12	Somalia	—	0	No	No
13	Somalia	—	1 (1)	No	Yes
14	Somalia	—	0	Yes	No
15	Norway	Pakistani	3 (1)	No	No
16	Pakistan	—	1 (0.5)	Yes	No

### Data Collection and Analysis

The study had a qualitative, explorative design to gain a deeper insight into perceptions about a culturally sensitive intervention. Individual and focus group interviews were conducted by the first author, either at the woman’s home, shelter, or in a private room at the university. The interviews followed a semi-structured guide and the main themes were as follows: (a) perceptions of the questions about violence and safety behaviors; (b) perceptions of what information a video about violence and safety behaviors should contain; and (c) perceptions of the draft video. Two pilot interviews were conducted to test the effectiveness of the interview guide and minor changes (such as suggestions based on asking what the video should contain before seeing it and the importance of translated questionnaires and videos to Urdu, Somali, and English) were undertaken. The two interviews are included in the data. All of the interviews lasted between 45 and 90 min, were audiotaped and transcribed verbatim.

The analysis of the interviews was guided by a thematic analysis strategy (Braun & Clarke, 2006). In search of patterns and noteworthy findings, the analysis was inductive and included five steps: (a) familiarization with the data by repeated reading of the transcripts, (b) generation of initial codes, (c) organization of codes into subthemes, (d) arrangement of subthemes into overarching themes, and (e) definition and naming of the themes. The program OpenCode (version 4.2.3) was used when generating initial codes and organizing codes into subthemes. The first author performed the preliminary analysis, followed by a discussion of potential themes and subthemes with the co-authors to strengthen the trustworthiness of the findings.

### Ethical Considerations

The Regional Committee for Medical and Health Research Ethics (REC) approved the study (ref.nr: 2017/358) and the interviews followed the Helsinki Protocol (World Medical Association [WMA], 2013) and ethical guidelines by the WHO (1999). Participants received verbal and written information about the purpose of the study and written consent was obtained from all participants. Women were informed that they would not be asked questions about IPV experiences. However, sensitive questions and a video about IPV might evoke memories and cause emotional distress. The interviewer made an effort to create a calm atmosphere and have an empathetic, nondemanding, and non-judgmental attitude during the interviews. The women had a friend or a family member to talk to after the interview, if required. In addition, the women were given the option to call the researcher.

## Results

Table 2 provides an overview of the results from the women’s feedback on the development of the Safe Pregnancy intervention. The participants’ feedback regarding questions about violence and safety behaviors resulted in two main themes. First, *perceptions of questions about IPV and safety behaviors* illustrates whether participants perceive the questions as relevant and easy to understand linguistically. However, the questions are sensitive and may prevent disclosure of IPV. *Facilitators and barriers to disclose IPV via a tablet* elucidated several aspects that participants thought were important for women to feel safe when disclosing violence. The importance of anonymity emerged as an essential prerequisite for disclosing violence. From the analysis of the participants’ perceptions of a video about violence and safety behaviors two main themes emerged. One, *perceptions of the information in the video about IPV and safety behaviors*, describes the importance of focusing on information about different forms of violence, safety-promoting behaviors, and help-seeking strategies. Second, *perceptions of the tools used in the video about IPV and safety behaviors* illuminates the desire for the tools to be perceived as stress reducing, helpful, and promoting the safety behaviors to be remembered.

**Table 2.** Women’s Feedback on the Development of the Safe Pregnancy Intervention.

Main theme	Subthemes
Perceptions of questions about IPV and safety behaviors	• Women perceived the questions to be sensitive but easy to understand • Women perceived the questions to be relevant
Facilitators and barriers to disclose IPV via a tablet	• Important to receive information about anonymity • Trust in the midwife to facilitate disclosure • Fear of consequences as barriers for disclosure • Positive with privacy when answering
Perceptions of the information in the video about IPV and safety behaviors	• Essential to inform about the different forms of IPV and its consequences • Important to inform about strategies for women to stay safe and to get help
Perceptions of the tools used in the video about IPV and safety behaviors	• Women perceived the voice and background music as stress reducing • Women found the pictures and illustrations to be helpful • Women valued the repetition of safety behaviors

*Note.* IPV = Intimate Partner Violence.

### Perceptions of Questions about IPV and Safety Behaviors

The first theme illustrates how women perceived the questions about IPV and safety behaviors. All participants said that the questions about violence and safety behaviors were easy to understand; however, they perceived the questions to be sensitive. A Norwegian woman suggested changing the order of the questions about violence. She emphasized that all of the questions are sensitive but that it might be easier for study participants to disclose IPV if the first questions address milder forms of violence:

. . . that you start with the mild and then it may be easier to be brave to answer honestly . . . maybe I would start with milder forms of violence like “followed me, harassed me, told me I was crazy, stupid, or not good enough.” (Participant 5)

Although the women identified the questions as sensitive, they said that the questions were relevant. Independent of their ethnic backgrounds, women revealed experiences with the acts of violence and the use of safety behaviors mentioned in the questionnaire:

A lot of this has happened to me. . . . I received help at a crisis shelter because I called the police. I had a code on my phone . . . with the neighbor. (Participant 3)

Based on participants’ feedback, we changed the order of the questions about violence, addressing milder forms of violence first. The women pointed out that the new order of the questions about violence was appropriate and that it could possibly facilitate disclosure of IPV.

### Facilitators and Barriers to Disclose IPV via a Tablet

The women were asked what would encourage them to disclose experiences with IPV via a tablet. The importance of anonymity was the most common subtheme in this theme. Independent of ethnicity and former experiences of IPV, women highlighted information about anonymity as a prerequisite for disclosure. A common statement from the women was:

. . . it (the answers) has to be anonymous . . . if you answer and know that the people at the health center can find out . . . but if your husband cannot see the answers, you may give honest answers. (Participant 2)

Some of the women emphasized the importance of repeating information about anonymity in the different sections of the questionnaire. A Somalian woman stated the following:

. . . it is difficult to answer . . . the questions are sensitive. Therefore it is important that you give information about anonymity at the start . . . and again before the questions about violence and safety behaviors. (Participant 10)

Despite information about anonymity, women with an immigrant background particularly expressed doubts regarding the trustworthiness of the information about anonymity. A woman originating from Pakistan expressed her skepticism in the following:

. . . is it anonymous? Why does she (the midwife) ask me? Does she (the midwife) know something? (Participant 15)

For women with an immigrant background, the assurance that their answers will be handled confidentially did not seem to be enough to disclose experiences of violence. In addition to written information about anonymity in the questionnaire, the women stated that oral information about anonymity from a midwife they trust might facilitate disclosure:

. . . the questions about violence and safety behaviors cannot be answered honestly. She must know that she is anonymous to answer honestly and you must create trust first through the midwife. (Participant 6)

Despite the common skepticism regarding trust in anonymity, another woman from Pakistan outlined that the use of the tablet could promote this trust:

. . . yes, definitely, because you are not writing your name, you are not scared. You can say: “No, I’m not that person who answered this.” (Participant 16)

While all women suggested important facilitators for IPV disclosure, they also highlighted potential barriers. All the women in our study emphasized that it would be easier to answer questions about violence and safety behaviors honestly, if their disclosure would not lead to serious consequences. A Norwegian woman with a history of IPV stated:

. . . then (living in a violent relationship), I would not have dared to answer the questions about violence honestly. If you feel that it does not have any consequences, you might answer honestly and it would be liberating and helpful. (Participant 2)

Independent of ethnicity, a common fear was that a violent partner would get to know about their disclosure and that it would expose the women and their children to further harm. In addition, some women expressed fear of shame as a barrier for revealing a violent relationship. Women with an immigrant background described a more complex situation addressing culturally sensitive aspects:

. . . and it is this talk about honor: “what would people say about me?,” “what would people think about what I say?” and “what would happen to my children?” (Participant 15)

The Somalian women expressed the difficulties for women from their culture to disclose violence as they fear that Child Welfare Services would take their children away from them.

All of the participants stated that being in a room alone while answering the questions could promote disclosure of violence. A common statement described by a woman originating from Pakistan was:

. . . if you ask someone while sitting in front of them, then she will definitely not (answer) but alone, she can (answer). (Participant 16)

However, the participants expressed that the questions about IPV and safety behaviors could cause emotional distress. Most participants said that the information about encouraging women to speak with their midwife if they have any concerns after filling out the questionnaire was important to promote safety.

Due to participant feedback, information about anonymity was given at the start of the questionnaire and repeated before the questions about IPV and safety behaviors. In accordance with the Safe Pregnancy study guidelines, women were alone while filling out the questionnaire.

### Perceptions of the Information in the Video About IPV and Safety Behaviors

Before seeing the draft video, participants provided varying advice on what information a video about safety behaviors should contain. Irrespective of ethnicity and experiences of violence, all participants emphasized that the video should contain information about different forms of violence. A common statement expressed by a woman originating from Norway was:

. . . it should not solely be about those who need to go to the emergency room. It should include psychological violence as well. (Participant 5)

Most women from immigrant backgrounds said that violence is accepted in their culture and that different forms of psychological violence are interpreted as normal behavior. They highlighted the importance of informing participants about what violence is, that violence is illegal in Norway, and that women suffering violence are victims. A Pakistani woman with a history of IPV said:

. . . it’s important that the girls know what violence is and that they do not feel that they are alone in being controlled, isolated, being the slave for the in-laws and being exposed to physical and psychological violence. (Participant 6)

After seeing the draft video, the women said that the information about physical violence was relevant and good. However, women with a history of psychological violence stated that the information was inadequate and failed to address women who only experienced psychological abuse:

. . . I don’t pay attention, because this does not apply to me. It is this to be partly isolated, partly deprived of the opportunity to spend your money and such. . . . Such things had been helpful to shed light on. (Participant 2)

Another woman originating from Norway shared this opinion. In addition, she pointed out the challenge of providing culturally sensitive information about safety behaviors that all women suffering violence can relate to:

. . . why would I need a passport? I’m not going anywhere. So you can think that this is not about you, but somebody else. (Participant 5)

Some women expressed that they missed information regarding how living in a violent environment could potentially affect their children. A woman explained that information facilitating awareness about the abnormality of violence could make it easier for women to disclose violence and ask for help:

. . . I wanted to know a little more about what could happen to the child when living in a violent relationship. You hope that when the child comes then it will be better and that the family is gathered and . . . that is what makes it so difficult to leave the partner . . . but you also want to put the children first . . . it might be easier to ask for help then. (Participant 3)

The participants highlighted information about what women exposed to violence can do to promote their safety. They presented a variety of suggestions, such as getting hold of their passport, hiding money, and making plans if they have to leave. Most women, independent of ethnicity and experiences of violence, stated that the most important safety behavior would be to talk to someone they trust. A woman originating from Norway outlined why:

. . . I think that the most important safety behavior would be that you dared to talk to someone. Few women will be able to get out (leave the violent partner) alone without help. (Participant 5)

The participants outlined the importance for women living in a violent relationship to receive information about where to get help. They expressed common suggestions such as having websites and phone numbers for the police and shelters. Women with immigrant backgrounds described a more complex situation:

They need information about where to get help. If you were born and raised here, you know your rights. But is it one who comes from abroad and is completely unknown, has no family, no chance to get out, it is very difficult. (Participant 7)

Resulting from participants’ feedback, the video was expanded to include more information about psychological abuse and potential consequences for children living in a violent environment.

### Perceptions of the Tools Used in the Video About IPV and Safety Behaviors

This theme concerns how women perceived the tools used in the video. All women emphasized that the sound, including the background music and the narrator’s voice, was perceived as calming, thus reducing emotional distress. A Norwegian woman expressed this common experience as follows:

The voice (the narrator) was clear and to the point. She (the narrator) spoke calmly and the music was comfortable, as comfortable as possible in relation to that message (sensitive) . . . I think it was nice that there was music . . . it reduces the . . . (emotional distress) . . . it (the music) makes the information about a sensitive topic less sensitive. (Participant 11)

The clear, calm voice and soft music made it easy to concentrate and receive the message. A Pakistani woman elaborated on this feedback:

. . . the soothing music in the background, it makes you think and consider (about the violent situation). (Participant 15)

The majority of the participants experienced the pictures in the video as informative and easy to understand and relate to. They highlighted that the pictures illustrated different forms of violence and the safety behaviors were perceived as relevant and helpful. In addition, the participants emphasized that the pictures about safety behaviors illustrated by women with different ethnic backgrounds might facilitate identification across cultural differences. A Somalian woman gave important feedback on this aspect:

. . . I like that you use pictures with different people . . . not everyone looks the same. Everyone is not Norwegian, blonde with blue eyes. I can see myself, a woman with a scarf on her head holding money in her hand. I think: “she is smart” and I can do the same (hide some money). (Participant 8)

Although most of the pictures were informative and easy to understand, the pictures illustrating psychological violence were perceived as confusing. A Norwegian woman with a history of IPV explained the challenge of illustrating psychological violence:

. . . I did not quite understand the picture illustrating psychological violence. I think that it does not belong here. I think about how I can illustrate it, and it is not easy to say. It is difficult to find images of psychological violence and hence understand what psychological violence is. (Participant 3)

Some women with different ethnic backgrounds, including professional participants from one focus group, suggested using a film with actors instead of static pictures in the video. Despite divergent opinions, most women stated that pictures, to a larger extent than film, can help abused women to identify with the sensitive information in the video. They stated that pictures promoted a quiet atmosphere that reduced emotional distress, and as a result, abused women would be more likely to remember the information in the video. A woman of Norwegian origin communicated common experiences related to the advantage of using a digital narrative with pictures instead of film:

. . . I think it is easier to pay attention when you see pictures . . . that there are no real people, because then it might be easier to relate it (the information) to themselves. . . . I think that it (pictures) might give more room for reflection and thoughts . . . and that it does not go too fast, because they (abused women) can get very occupied in their own experiences and then you will not pay attention to what is happening. (Participant 11)

Due to the sensitive topic, the women acknowledged that study participants experiencing IPV might suffer emotional distress when watching the video. Hence, they said that it was important to repeat information about safety behaviors at the end of the video.

. . . the narrator repeated several times the safety behaviors, and I believe that it is important. The video may cause many thoughts and then you may not hear what is said the first time. (Participant 11)

Based on the findings from this study, a short film illustrating psychological violence was included in the video. All other pictures and illustrations were kept. The focus group participants said that the adjusted film illustrated psychological violence well.

## Discussion

This study provided important insights to develop a culturally sensitive intervention to promote the prevention of IPV. Our findings show that women are in favor of disclosing IPV via a tablet. They expressed various suggestions about how to address barriers for disclosure, such as safeguarding anonymity and creating a trustful relationship with the midwife.

Women in this study were positive about answering questions on violence via a tablet at a MCHC. This is in line with results from prior studies documenting that use of electronic devices may eliminate many of the barriers associated with face-to-face screening (Bacchus et al., 2016; Klevens et al., 2012; Koziol-McLain et al., 2018; Taft et al., 2015). In a study of pregnant women in obstetric clinics in Australia comparing women’s preferences of in-person versus computer screening for IPV, participants were more likely to disclose IPV via a computer mainly due to anonymity (Chang et al., 2012). Similar to these results, women in our study stated that anonymity would be crucial for disclosing IPV. This is supported by others. Bacchus et al. (2016) conducted a technology-based IPV intervention in perinatal home visitation among women experiencing IPV in the United States. An intervention strategy on a computer tablet was perceived as a safe and confidential tool that appeared to offer women a greater sense of anonymity and privacy, thereby encouraging more openness in answering questions about violence (Bacchus et al., 2016).

However, the majority of women in our study with immigrant backgrounds said that they did not trust the tablet to safeguard their anonymity. Similar to Chang et al. (2012), they were uncertain who would be getting the information from their IPV disclosure on the tablet. This is in line with a qualitative study of Somali-born refugees in Sweden which revealed that questions about violence were met with hesitance (Byrskog et al., 2016). A key finding in our study was that decisions for and against disclosure of IPV often depend on a trusting relationship with the midwife. This echoes findings in prior qualitative studies which explore facilitators for IPV disclosure (Bacchus et al., 2016; G. S. Feder et al., 2006; Garnweidner-Holme et al., 2017; Spangaro et al., 2016, 2020). Spangaro et al. (2016) studied Aboriginal Australian women’s decisions to disclose IPV during pregnancy. They found that cultural safety was important for Aboriginal women and that building a trusting relationship was part of the process of creating cultural safety (Spangaro et al., 2016).

Previous research has revealed that women do not always disclose experiences of IPV and the prevalence may be underreported (Evans & Feder, 2016; Garcia-Moreno et al., 2015). Despite women in our study being in favor of disclosing abuse via a tablet, fear of consequences was a central finding that could prevent them from disclosing. Similar to our study, a qualitative study among multicultural women in south eastern Norway who experienced violence during pregnancy found that fear of their partner was one of the main barriers to disclosure (Garnweidner-Holme et al., 2017). In addition, women in our study from immigrant backgrounds highlighted fear of Child Welfare Services as an important barrier to disclosure for women from their culture. This is in line with results from other qualitative and quantitative studies from other countries (Alaggia et al., 2012; Byrskog et al., 2016; Foronda, 2008; Garnweidner-Holme et al., 2017; Ogunsiji et al., 2012; Spangaro et al., 2020), which document that cultural barriers can impede immigrant women’s opportunities and ability to disclose experiences of IPV.

Women in our study believed that the safety-promoting video might prevent IPV. However, they asked for more information about psychological violence. Similar to findings in a study by Young-Hauser et al. (2014), women in our study feared that failing to address psychological abuse could potentially alienate women who only experienced psychological abuse and exclude them from using the safety behaviors and thus prevent IPV. Women from immigrant backgrounds, in particular, said that harassment and controlling behavior have been accepted and normalized in their culture. This finding is in agreement with previous research focusing on migrant and minority women and IPV, suggesting this reflects cultural attitudes to IPV (Nash, 2005; Ogunsiji et al., 2012; Ting & Panchanadeswaran, 2009).

Qualitative and quantitative studies among abused women from different ethnicities have found that concerns for their children can be an important incentive to seeking help (Chang et al., 2010; Fanslow & Robinson, 2010; Ting & Panchanadeswaran, 2009). Women in our study supported this finding. They highlighted that concerns for the safety and well-being of their children would be important information in the video and would help abused women disclose violence and leave a violent partner. Hence, this was emphasized in the video.

Emotional reactions regarding the sensitive content of the tools used in the video seemed to be unavoidable. Women in our study acknowledged that the video would potentially trigger painful memories, and they expressed important views on the different tools aiming to minimize their emotional impact and ensure abused women’s well-being. Similar to the findings in a qualitative study aiming to modify an internet-based safety decision aid to a New Zealand context (Young-Hauser et al., 2014), we facilitated the well-being of women by using tools that might minimize emotional distress, such as the narrator’s calm voice, soothing music, and static pictures.

### Limitations and Methodological Considerations

Our study has limitations. Due to a qualitative study design aiming to gain a deeper understanding about a phenomenon, the study sample is too small to draw conclusions about a broader population of women (Braun & Clarke, 2006). However, the results are derived from women’s reported experiences and may be transferrable to similar groups. There was an imbalance among women who had experienced IPV and those who had not, but all women gave detailed and information-rich descriptions about the research questions. Women experiencing IPV were recruited from crisis shelters and not from a MCHC where the Safe Pregnancy study is conducted. Despite this, they could relate to the MCHC setting because they had children, thus experiencing antenatal care checkups at the MCHC in the past. Even though the interviews did not take place in a MCHC, the context was largely identical to the Safe Pregnancy study guidelines (Henriksen et al., 2019). To strengthen trustworthiness, essential measures for others to judge the value of this study have been taken throughout the research process. These include the use of an established and clearly described method for data analysis (Braun & Clarke, 2006) and the use of women’s quotations which facilitate the readers’ evaluation of reliability. All authors read the data and participated in the analysis of defining themes and subthemes until consensus was achieved (Braun & Clarke, 2006).

## References

[bibr1-1077801220954274] Alaggia R. Regehr C. Jenney A. (2012). Risky business: An ecological analysis of intimate partner violence disclosure. Research on Social Work Practice, 22, 301–312. 10.1177/1049731511425503

[bibr2-1077801220954274] Alhusen J. L. Ray E. Sharps P. Bullock L. (2015). Intimate partner violence during pregnancy: Maternal and neonatal outcomes. Journal of Women’s Health, 24, 100–106. 10.1089/jwh.2014.4872 PMC4361157 25265285

[bibr3-1077801220954274] Bacchus L. J. Bullock L. Sharps P. Burnett C. Schminkey D. L. Buller A. M. Campbell J. (2016). Infusing technology into perinatal home visitation in the United States for women experiencing intimate partner violence: Exploring the interpretive flexibility of an mHealth intervention. Journal of Medical Internet Research, 18, Article e302. 10.2196/jmir.6251 PMC5133433 27856405

[bibr4-1077801220954274] Braun V. Clarke V. (2006). Using thematic analysis in psychology. Qualitative Research in Psychology, 3, 77–101.

[bibr5-1077801220954274] Brownridge D. A. Taillieu T. L. Tyler K. A. Tiwari A. Ko Ling C. Santos S. C. (2011). Pregnancy and intimate partner violence: Risk factors, severity, and health effects. Violence Against Women, 17, 858–881. 10.1177/1077801211412547 21775311

[bibr6-1077801220954274] Burnett C. Bacchus L. (2016). Women find safety planning more useful than referrals in a maternal and child health IPV intervention. Evidence Based Nursing, 19, Article 43. 10.1136/eb-2015-102201 26612246

[bibr7-1077801220954274] Byrskog U. Essen B. Olsson P. Klingberg-Allvin M. (2016). “Moving on” violence, wellbeing and questions about violence in antenatal care encounters. A qualitative study with Somali-born refugees in Sweden. Midwifery, 40, 10–17. 10.1016/j.midw.2016.05.009 27428093

[bibr8-1077801220954274] Chang J. C. Dado D. Hawker L. Cluss P. A. Buranosky R. Slagel L. McNeil M. Scholle S. H. (2010). Understanding turning points in intimate partner violence: Factors and circumstances leading women victims toward change. Journal of Women’s Health, 19, 251–259. 10.1089/jwh.2009.1568 PMC2834452 20113147

[bibr9-1077801220954274] Chang J. C. Dado D. Schussler S. Hawker L. Holland C. L. Burke J. G. Cluss P. A. (2012). In person versus computer screening for intimate partner violence among pregnant patients. Patient Education and Counseling, 88, 443–448. 10.1016/j.pec.2012.06.021 22770815 PMC3413751

[bibr10-1077801220954274] Devries K. M. Kishor S. Johnson H. Stockl H. Bacchus L. J. Garcia-Moreno C. Watts C. (2010). Intimate partner violence during pregnancy: Analysis of prevalence data from 19 countries. Reproductive Health Matters, 18, 158–170. 10.1016/S0968-8080(10)36533-5 21111360

[bibr11-1077801220954274] Edin K. E. Dahlgren L. Lalos A. Högberg U. (2010). “Keeping up a front”: Narratives about intimate partner violence, pregnancy, and antenatal care. Violence Against Women, 16, 189–206. 10.1177/1077801209355703 20053947

[bibr12-1077801220954274] Eustace J. Baird K. Saito A. S. Creedy D. K. (2015). Midwives’ experiences of routine enquiry for intimate partner violence in pregnancy. Women and Birth, 29, 503–510. 10.1016/j.wombi.2016.04.010 27178111

[bibr13-1077801220954274] Evans M. A. Feder G. S. (2016). Help-seeking amongst women survivors of domestic violence: A qualitative study of pathways towards formal and informal support. Health Expect, 19, 62–73. 10.1111/hex.12330 25556776 PMC5055220

[bibr14-1077801220954274] Fanslow J. L. Robinson E. M. (2010). Help-seeking behaviors and reasons for help seeking reported by a representative sample of women victims of intimate partner violence in New Zealand. Journal of Interpersonal Violence, 25, 929–951. 10.1177/0886260509336963 19597160

[bibr15-1077801220954274] Feder G. Ramsay J. Dunne D. Rose M. Arsene C. Norman R. Kuntze S. Spencer A. Bacchus L. Hague G. Warburton A. Taket A. (2009). How far does screening women for domestic (partner) violence in different health-care settings meet criteria for a screening programme? Systematic reviews of nine UK National Screening Committee criteria. Health Technology Assessment, 13, iii–iv, xi–xiii, 1–113, 137–347. 10.3310/hta13160 19272272

[bibr16-1077801220954274] Feder G. S. Hutson M. Ramsay J. Taket A. R. (2006). Women exposed to intimate partner violence: Expectations and experiences when they encounter health care professionals: A meta-analysis of qualitative studies. Archives of Internal Medicine, 166, 22–37. 10.1001/archinte.166.1.22 16401807

[bibr17-1077801220954274] Finnbogadottir H. Dykes A.-K. (2016). Increasing prevalence and incidence of domestic violence during the pregnancy and one and a half year postpartum, as well as risk factors: A longitudinal cohort study in Southern Sweden. BMC Pregnancy Childbirth, 16, Article 327. 10.1186/s12884-016-1122-6 PMC5081903 27784283

[bibr18-1077801220954274] Finnbogadottir H. Dykes A.-K. Wann-Hansson C. (2014). Prevalence of domestic violence during pregnancy and related risk factors: A cross-sectional study in southern Sweden. BMC Women’s Health, 14, Article 63. 10.1186/1472-6874-14-63 PMC4030020 24885532

[bibr19-1077801220954274] Ford-Gilboe M. Wathen C. N. Varcoe C. MacMillan H. L. Scott-Storey K. Mantler T. Hegarty K. Perrin N. (2016). Development of a brief measure of intimate partner violence experiences: The Composite Abuse Scale (Revised)-Short Form (CASR-SF). BMJ Open, 6, Article e012824. 10.1136/bmjopen-2016-012824 PMC5168640 27927659

[bibr20-1077801220954274] Foronda C. L. (2008). A concept analysis of cultural sensitivity. Journal of Transcultural Nursing, 19, 207–212. 10.1177/1043659608317093 18411414

[bibr21-1077801220954274] Garcia-Moreno C. Hegarty K. d’Oliveira A. F. L. Koziol-McLain J. Colombini M. Feder G. S. (2015). The health-systems response to violence against women. Lancet, 385, 1567–1579. 10.1016/S0140-6736(14)61837-7 25467583

[bibr22-1077801220954274] García-Moreno C. Pallitto C. Devries K. Stöckl H. Watts C. Abrahams N. Petzold M. (2013). Global and regional estimates of violence against women: Prevalence and health effects of intimate partner violence and non-partner sexual violence. https://www.who.int/iris/bitstream/10665/85239/1/9789241564625_eng.pdf

[bibr23-1077801220954274] Garnweidner-Holme L. M. Lukasse M. Solheim M. Henriksen L. (2017). Talking about intimate partner violence in multi-cultural antenatal care: A qualitative study of pregnant women’s advice for better communication in South-East Norway. BMC Pregnancy and Childbirth, 17, Article 123. 10.1186/s12884-017-1308-6 PMC5395889 28420328

[bibr24-1077801220954274] Gidman J. (2013). Listening to stories: Valuing knowledge from patient experience. Nurse Education in Practice, 13, 192–196. 10.1016/j.nepr.2012.09.006 23084838

[bibr25-1077801220954274] Hawkins R. P. Kreuter M. Resnicow K. Fishbein M. Dijkstra A. (2008). Understanding tailoring in communicating about health. Health Education Research, 23, 454–466. 10.1093/her/cyn004 18349033 PMC3171505

[bibr26-1077801220954274] Hegarty K. Tarzia L. Murray E. Valpied J. Humphreys C. Taft A. Gold L. Glass N. (2015). Protocol for a randomised controlled trial of a web-based healthy relationship tool and safety decision aid for women experiencing domestic violence (I-DECIDE). BMC Public Health, 15, Article 736. 10.1186/s12889-015-2072-z PMC4522060 26231225

[bibr27-1077801220954274] Helsedirektoratet. (2014). Nasjonal faglig retningslinje for svangerskapsomsorgen—hvordan avdekke vold [National guidelines for antenatal care in Norway-how to uncover violence]. https://bufdir.no/bibliotek/Dokumentside/?docId=BUF00002432

[bibr28-1077801220954274] Helsedirektoratet. (2019). Svangerskapsomsorgen: Nasjonal faglig retningslinje [National guidelines for antenatal care in Norway]. https://www.helsedirektoratet.no/retningslinjer/svangerskapsomsorgen

[bibr29-1077801220954274] Henriksen L. Flaathen E. M. Angelshaug J. Garnweidner-Holme L. Smastuen M. C. Noll J. Taft A. Schei B. Lukasse M. (2019). The Safe Pregnancy study—Promoting safety behaviours in antenatal care among Norwegian, Pakistani and Somali pregnant women: A study protocol for a randomized controlled trial. BMC Public Health, 19, Article 724. 10.1186/s12889-019-6922-y PMC6558870 31182062

[bibr30-1077801220954274] Henriksen L. Garnweidner-Holme L. I. M. Thorsteinsen K. K. Lukasse M. (2017). “It is a difficult topic”—A qualitative study of midwives’ experiences with routine antenatal enquiry for intimate partner violence. BMC Pregnancy and Childbirth, 17, Article 165. 10.1186/s12884-017-1352-2 PMC5457554 28577361

[bibr31-1077801220954274] Henriksen L. Schei B. Vangen S. Lukasse M. (2014). Sexual violence and mode of delivery: A population-based cohort study. BJOG: An International Journal of Obstetrics and Gynaecology, 121, 1237–1244. 10.1111/1471-0528.12923 24939396

[bibr32-1077801220954274] Henriksen L. Vangen S. Schei B. Lukasse M. (2013). Sexual violence and antenatal hospitalization. Birth, 40, 281–288. 10.1111/birt.12063 24344709

[bibr33-1077801220954274] Hill A. Pallitto C. McCleary-Sills J. Garcia-Moreno C. (2016). A systematic review and meta-analysis of intimate partner violence during pregnancy and selected birth outcomes. International Journal of Gynecology and Obstetrics, 133, 269–276. 10.1016/j.ijgo.2015.10.023 27039053

[bibr34-1077801220954274] Hjemdal O. K. Engnes K. (2009). Å spørre om vold ved svangerskapskontroll (Rapport 1/2009) [Screening for violence during prenatal checkups:A trial in four Norwegian municipalities]. https://www.nkvts.no/rapport/a-sporre-om-vold-ved-svangerskapskontroll-rapport-fra-et-forsoksprosjekt-i-fire-kommuner/

[bibr35-1077801220954274] Hooker L. Samaraweera N. Y. Agius P. A. Taft A. (2016). Intimate partner violence and the experience of early motherhood: A cross-sectional analysis of factors associated with a poor experience of motherhood. Midwifery, 34, 88–94. 10.1016/j.midw.2015.12.011 26805605

[bibr36-1077801220954274] Jahanfar S. Janssen P. A. Howard L. M. Dowswell T. (2013). Interventions for preventing or reducing domestic violence against pregnant women. Cochrane Database of Systematic Reviews, 2, Article CD009414. 10.1002/14651858.CD009414.pub2 23450603

[bibr37-1077801220954274] James L. Brody D. Hamilton Z. (2013). Risk factors for domestic violence during pregnancy: A meta-analytic review. Violence and Victims, 28, 359–380. 23862304 10.1891/0886-6708.vv-d-12-00034

[bibr38-1077801220954274] Kiely M. El-Mohandes A. A. E. El-Khorazaty M. N. Blake S. M. Gantz M. G. (2010). An integrated intervention to reduce intimate partner violence in pregnancy: A randomized controlled trial. Obstetrics and Gynecology, 115, 273–283. 10.1097/AOG.0b013e3181cbd482 20093899 PMC2917915

[bibr39-1077801220954274] Klevens J. Sadowski L. Kee R. Trick W. Garcia D. (2012). Comparison of screening and referral strategies for exposure to partner violence. Women’s Health Issues, 22, e45–e52. 10.1016/j.whi.2011.06.008 21798763

[bibr40-1077801220954274] Koziol-McLain J. Vandal A. C. Wilson D. Nada-Raja S. Dobbs T. McLean C. Sisk R. Eden K. B. Glass N. E. (2018). Efficacy of a web-based safety decision aid for women experiencing intimate partner violence: Randomized controlled trial. Journal of Medical Internet Research, 20, Article e8. 10.2196/jmir.8617 PMC6858022 29321125

[bibr41-1077801220954274] Kreuter M. W. Lukwago S. N. Bucholtz R. D. D. C. Clark E. M. Sanders-Thompson V. (2003). Achieving cultural appropriateness in health promotion programs: Targeted and tailored approaches. Health Education & Behavior, 30, 133–146. 10.1177/1090198102251021 12693519

[bibr42-1077801220954274] Kreuter M. W. McClure S. M. (2004). The role of culture in health communication. Annual Review of Public Health, 25, 439–455. 10.1146/annurev.publhealth.25.101802.123000 15015929

[bibr43-1077801220954274] LeRouge C. Ma J. Sneha S. Tolle K. (2013). User profiles and personas in the design and development of consumer health technologies. International Journal of Medical Informatics, 82, e251–e268. 10.1016/j.ijmedinf.2011.03.006 21481635

[bibr44-1077801220954274] Lukasse M. Schroll A.-M. Ryding E. L. Campbell J. Karro H. Kristjansdottir H. Laanpere M. Steingrimsdottir T. Tabor A. Temmerman M. Van Parys A.-S. Wangel A.-M. Schei B. (2014). Prevalence of emotional, physical and sexual abuse among pregnant women in six European countries. Acta Obstetricia et Gynecologica Scandinavica, 93, 669–677. 10.1111/aogs.12392 24720803

[bibr45-1077801220954274] McFarlane J. Malecha A. Gist J. Watson K. Batten E. Hall I. Smith S. (2004). Increasing the safety-promoting behaviors of abused women. American Journal of Nursing, 104, 40–50; quiz 50–41. 10.1097/00000446-200403000-00019 15108570

[bibr46-1077801220954274] McFarlane J. M. Groff J. Y. O’Brien J. A. Watson K. (2006). Secondary prevention of intimate partner violence: A randomized controlled trial. Nursing Research, 55, 52–61. 16439929 10.1097/00006199-200601000-00007

[bibr47-1077801220954274] Moonesinghe L. N. Rajapaksa L. C. Samarasinghe G. (2004). Development of a screening instrument to detect physical abuse and its use in a cohort of pregnant women in Sri Lanka. Asia-Pacific Journal of Public Health, 16, 138–144. 10.1177/101053950401600211 15624793

[bibr48-1077801220954274] Nash S. T. (2005). Through Black eyes: African American women’s constructions of their experiences with intimate male partner violence. Violence Against Women, 11, 1420–1440. 10.1177/1077801205280272 16204732

[bibr49-1077801220954274] O’Doherty L. J. Taft A. Hegarty K. Ramsay J. Davidson L. L. Feder G. (2014). Screening women for intimate partner violence in healthcare settings: Abridged Cochrane systematic review and meta-analysis. British Medical Journal, 348, Article g2913. 10.1136/bmj.g2913 PMC4018471 24821132

[bibr50-1077801220954274] Ogunsiji O. Wilkes L. Jackson D. Peters K. (2012). Suffering and smiling: West African immigrant women’s experience of intimate partner violence. Journal of Clinical Nursing, 21, 1659–1665. 10.1111/j.1365-2702.2011.03947.x 22151055

[bibr51-1077801220954274] Pallitto C. Garcia-Moreno C. Stoeckl H. Hatcher A. MacPhail C. Mokoatle K. Woollett N. (2016). Testing a counselling intervention in antenatal care for women experiencing partner violence: A study protocol for a randomized controlled trial in Johannesburg, South Africa. BMC Health Services Research, 16, Article 630. 10.1186/s12913-016-1872-x PMC5097399 27814706

[bibr52-1077801220954274] Rabin R. F. Jennings J. M. Campbell J. C. Bair-Merritt M. H. (2009). Intimate partner violence screening tools: A systematic review. American Journal of Preventive Medicine, 36, 439–445, e434. 10.1016/j.amepre.2009.01.024 PMC2688958 19362697

[bibr53-1077801220954274] Resnicow K. Baranowski T. Ahluwalia J. S. Braithwaite R. L. (1999). Cultural sensitivity in public health: Defined and demystified. Ethnicity & Disease, 9, 10–21. 10355471

[bibr54-1077801220954274] Sorbo M. F. Grimstad H. Bjorngaard J. H. Schei B. Lukasse M. (2013). Prevalence of sexual, physical and emotional abuse in the Norwegian mother and child cohort study. BMC Public Health, 13, Article 186. 10.1186/1471-2458-13-186 PMC3599849 23452504

[bibr55-1077801220954274] Spangaro J. Herring S. Koziol-Mclain J. Rutherford A. Frail M.-A. Zwi A. B. (2016). “They aren’t really black fellas but they are easy to talk to”: Factors which influence Australian Aboriginal women’s decision to disclose intimate partner violence during pregnancy. Midwifery, 41, 79–88. 10.1016/j.midw.2016.08.004 27551857

[bibr56-1077801220954274] Spangaro J. Koziol-McLain J. Rutherford A. Zwi A. B. (2020). “Made me feel connected”: A qualitative comparative analysis of intimate partner violence routine screening pathways to impact. Violence Against Women, 26, 334–358. 10.1177/1077801219830250 30870117

[bibr57-1077801220954274] Sprague C. Hatcher A. M. Woollett N. Black V. (2017). How nurses in Johannesburg address intimate partner violence in female patients: Understanding IPV responses in low- and middle-income country health systems. Journal of Interpersonal Violence, 32, 1591–1619. 10.1177/0886260515589929 26092654

[bibr58-1077801220954274] Sprague S. Madden K. Simunovic N. Godin K. Pham N. K. Bhandari M. Goslings J. C. (2012). Barriers to screening for intimate partner violence. Women & Health, 52, 587–605. 10.1080/03630242.2012.690840 22860705

[bibr59-1077801220954274] Stenson K. Saarinen H. Heimer G. Sidenvall B. (2001). Women’s attitudes to being asked about exposure to violence. Midwifery, 17, 2–10. 10.1054/midw.2000.0241 11207100

[bibr60-1077801220954274] Taft A. J. Hooker L. Humphreys C. Hegarty K. Walter R. Adams C. Agius P. Small R. (2015). Maternal and child health nurse screening and care for mothers experiencing domestic violence (MOVE): A cluster randomised trial. BMC Medicine, 13, Article 150. 10.1186/s12916-015-0375-7 PMC4480893 26111528

[bibr61-1077801220954274] Ting L. Panchanadeswaran S. (2009). Barriers to help-seeking among immigrant African women survivors of partner abuse: Listening to women’s own voices. Journal of Aggression, Maltreatment, & Trauma, 18, 817–838. 10.1080/10926770903291795

[bibr62-1077801220954274] Tiwari A. Leung W. C. Leung T. W. Humphreys J. Parker B. Ho P. C. (2005). A randomised controlled trial of empowerment training for Chinese abused pregnant women in Hong Kong. BJOG: An International Journal of Obstetrics and Gynaecology, 112, 1249–1256. 10.1111/j.1471-0528.2005.00709.x 16101604

[bibr63-1077801220954274] Van Parys A.-S. Verhamme A. Temmerman M. Verstraelen H. (2014). Intimate partner violence and pregnancy: A systematic review of interventions. PLOS ONE, 9, Article e85084. 10.1371/journal.pone.0085084 PMC3901658 24482679

[bibr64-1077801220954274] Vatnar S. K. B. Bjorkly S. (2010). Does it make any difference if she is a mother? An interactional perspective on intimate partner violence with a focus on motherhood and pregnancy. Journal of Interpersonal Violence, 25, 94–110. 10.1177/0886260508329129 19150888

[bibr65-1077801220954274] World Health Organization. (1999). Putting women first: Ethical and safety recommendations for research on domestic violence against women. https://www.who.int/gender-equity-rights/knowledge/who_fch_gwh_01.1/en/

[bibr66-1077801220954274] World Health Organization. (2011). mHealth: New horizons for health through mobile technologies: Second global survey on eHealth. https://apps.who.int/iris/handle/10665/44607

[bibr67-1077801220954274] World Medical Association. (2013). WMA declaration of Helsinki—Ethical principles for medical research involving human subjects. https://www.wma.net/policies-post/wma-declaration-of-helsinki-ethical-principles-for-medical-research-involving-human-subjects/10.1001/jama.2013.281053 24141714

[bibr68-1077801220954274] Young-Hauser A. M. Eden K. B. Wilson D. Koziol-Mclain J. (2014). Intimate partner violence: Modifying an internet-based safety decision aid to a New Zealand context. Journal of Technology in Human Services, 32, 297–311. 10.1080/15228835.2014.967905

